# Photodynamic therapy with *Bixa orellana* extract and LED for the reduction of halitosis: study protocol for a randomized, microbiological and clinical trial

**DOI:** 10.1186/s13063-018-2913-z

**Published:** 2018-10-29

**Authors:** Marcela Leticia Leal Gonçalves, Ana Carolina Costa da Mota, Alessandro Melo Deana, Guelton Hirano Guedes, Lisyanne Araújo de Souza Cavalcante, Renato Araújo Prates, Anna Carolina Ratto Tempestini Horliana, Christiane Pavani, Lara Jansiski Motta, Greice de Brito Bitencourt, Kristianne Porta Santos Fernandes, Monica da Consolação Canuto Salgueiro, Raquel Agnelli Mesquita-Ferrari, Daniela Fátima Teixeira da Silva, Cristiane Miranda França, Sandra Kalil Bussadori

**Affiliations:** 1 Biophotonics Applied to Health Sciences, Universidade Nove de Julho, Vergueiro Street, 235/249, Liberdade, São Paulo, SP ZIP 01504-001 Brazil; 2 Universidade Nove de Julho, Vergueiro Street, 235/249, Liberdade, São Paulo, SP ZIP 01504-001 Brazil; 3 Rehabilitation Sciences, Universidade Nove de Julho, Vergueiro Street, 235/249, Liberdade, São Paulo, SP ZIP 01504-001 Brazil; 4 Biomaterials and Biomechanics, School of Dentistry, Oregon Health and Science University, 2730 S.W. Moody Ave, Portland, OR 97201 USA

**Keywords:** Halitosis, Photodynamic therapy, *Bixa orellana*

## Abstract

**Background:**

Halitosis is an unpleasant breath odour that can interfere with the professional life, social life and quality of life of people who suffer from it. A modality of treatment that has been increasing in dentistry is antimicrobial photodynamic therapy (aPDT). *Bixa orellana*, popularly known as “urucum” is a plant native to Brazil. The seeds are used to produce a dye that is largely used in the food, textile, paint and cosmetic industries. The aim of this study is to verify whether aPDT with *Bixa orellana* extract and blue light-emitting diodes (LEDs) is effective in reducing halitosis. This method will also be compared with tongue scraping, the most commonly used conventional method for tongue coating removal, and the association of both methods will be evaluated.

**Methods/design:**

A randomized clinical trial will be conducted at the dental clinic of the Universidade Nove de Julho*.* Thirty-nine patients will be divided by block randomization into three groups (*n* = 13) according to the treatment to be performed. In Group 1, tongue scraping will be performed by the same operator in all patients for analysis of the immediate results. Patients will also be instructed on how to use the scraper at home. Group 2 will be treated with aPDT with *Bixa orellana* extract and the LED light curing device: Valo Cordless Ultradent®. Six points in the tongue dorsum with a distance of 1 cm between them will be irradiated. The apparatus will be pre-calibrated at wavelength 395–480 nm for 20 s and 9.6 J per point. In Group 3, patients will be submitted to the tongue scraping procedure, as well as to the previously explained aPDT. Oral air collection with the Oral Chroma™ and microbiological collections of the tongue coating shall be done before, immediately after and 7 days after treatment for comparison.

**Discussion:**

Halitosis treatment is a topic that still needs attention. The results of this trial could support decision-making by clinicians regarding aPDT using blue LEDs for treating halitosis on a daily basis, as most dentists already have this light source in their offices.

**Trial registration:**

ClinicalTrials.gov, NCT03346460. Registered on 17 November 2017.

**Electronic supplementary material:**

The online version of this article (10.1186/s13063-018-2913-z) contains supplementary material, which is available to authorized users.

## Background

Halitosis is an unpleasant breath odour that can interfere with the professional life, social life and quality of life of people who suffer from it [1, 2]. The prevalence of this condition may greatly vary, depending on the population. Studies show that moderate halitosis affects one third of individuals [3]. Bad breath can have both extraoral and intraoral causes, such as the use of some types of medication, dry mouth, smoking, systemic conditions and inefficient oral hygiene [1, 4]. Particularly in young patients, the presence of tongue coating seems to be the main cause of halitosis [5]. To diagnose halitosis, some tests can be conducted [6]. Among them, the organoleptic method is very often used; however, it is considered a subjective measure. The use of the Oral Chroma™, on the other hand, is the most indicative way of determining the presence of halitosis, since it has the ability to distinguish between the volatile sulphur compounds (VSCs) responsible for halitosis. Additionally, it can determine not only the presence of halitosis, but also its origin, according to the most prevalent gases [7].

The most common treatments for halitosis include the use of mouthwashes, periodontal treatment, oral hygiene instructions and tongue coating cleaning [8, 9]. While the mechanical removal of tongue coating can be easily done by patients themselves and is widely recommended, it is an oral hygiene procedure that is little practiced due to discomfort or lack of awareness [10]. Regarding mouthwashes, some of them seem to be efficient in the temporary reduction of halitosis, but few data with respect to their efficiency in tongue coating reduction are available [11–13]. The combination of more than one treatment, such as tongue cleaning associated with periodontal treatment and rinses, always seems to have better results [13, 14], which indicates the complexity of the problem. Considering this, it is thus necessary to do more research in this area, introducing new techniques for the reduction of this condition.

A modality of treatment that has been increasing in dentistry is antimicrobial photodynamic therapy (aPDT). This therapy is based on the principle that the combination of a dye, called a photosensitizer (PS), with an appropriate wavelength light source and the ambient oxygen can produce reactive oxygen species that may lead to the death of microorganisms [10]. aPDT presents a series of advantages, such as unlikely development of resistance by bacteria and the fact that it is a non-invasive procedure [15]. Its use is being studied in several areas in dentistry, such as periodontics [16] and endodontics [17] and in the treatment of mucosa wounds [18] and caries [19]. aPDT has also been used in clinical trials for the reduction of halitosis with methylene blue and red lasers, and satisfactory results have been obtained [7, 20–22].

*Bixa orellana*, popularly known as “urucum”, is a plant native to Brazil. The seeds are used to produce a dye that is largely used in the food, textile, paint and cosmetic industries. This dye is one of the few that are accepted by the World Health Organization (WHO), due to the fact that it is not toxic [23]. It has important antioxidant and antimicrobial activities [24], and recent studies have shown its potential as a therapeutic agent, source of drugs and natural dye [23, 25]. Also, the use of red/yellowish dyes, such as curcumin, is being researched for their application in aPDT [26, 27]. Due to its positive characteristics, especially regarding the lack of mutagenic and cytotoxic activity, allied to the promising results reported by laboratory studies regarding its antimicrobial effect, *Bixa orellana* extract could be considered a suitable candidate as a PS. Besides that, most dentists already have blue light-emitting diodes (LEDs) in their offices, so using a red dye with these LEDs would make aPDT treatment more accessible.

### Hypothesis

Based on this background, this research will test the hypothesis that aPDT with *Bixa orellana* extract and blue LED is effective in the reduction of halitosis.

### Objective

The aim of this study is to verify whether aPDT with *Bixa orellana* extract and blue LED is effective in the reduction of halitosis, providing an accessible treatment for this condition, as the majority of dentists already have this sort of device in their offices. This method will also be compared with tongue scraping, the most commonly used conventional method for tongue coating removal, and the association of both methods will also be evaluated.

## Methods/design

### Study design

**Fig. 1:**
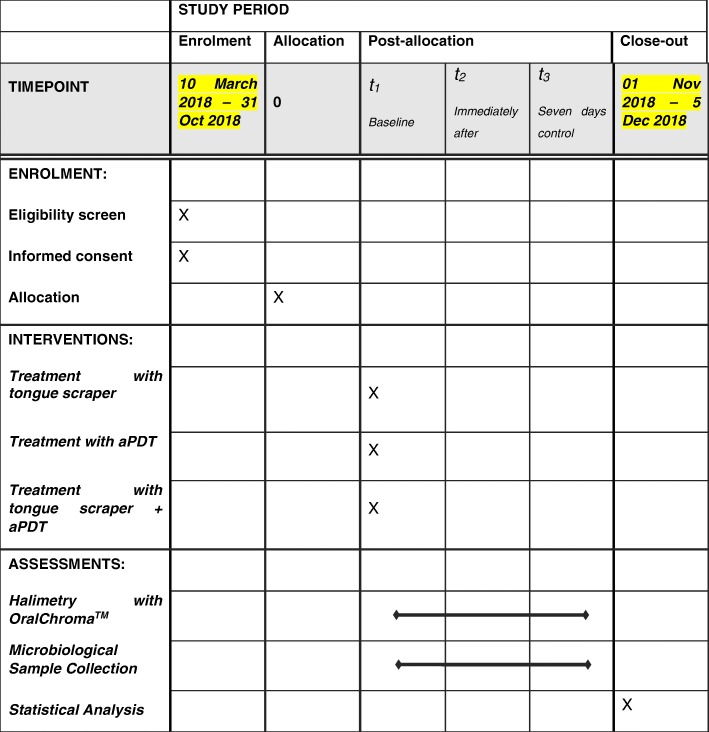
Schedule of enrolment, interventions and assessments of the present study

The protocol is in accordance with the 2013 Standard Protocol Items: Recommendations for Interventional Trials (SPIRIT) Statement. The SPIRIT checklist can be found as Additional file 1, and the schedule of enrolment, interventions and assessments of the present study is depicted in Fig. 1.

**Fig. 2:**
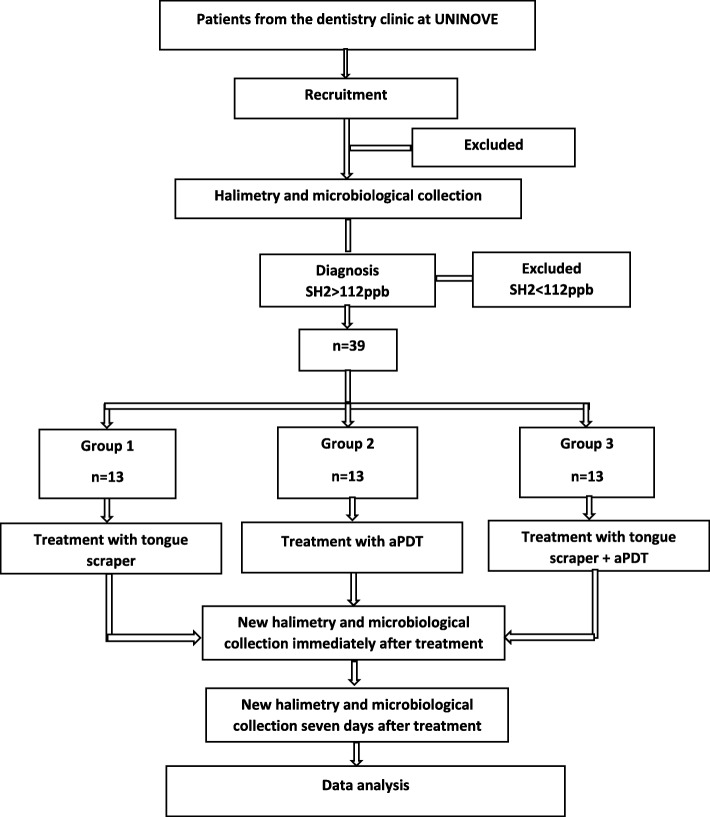
Trial activity flowchart

This study has been approved by the Ethics Committee of Universidade Nove de Julho under process number 74228417.2.0000.5511. Possible changes in this project shall be reported to the committee. All participants who agree to participate voluntarily will sign an informed consent form, as stipulated in Resolution 466/2012 of the Brazilian National Board of Health. A randomized clinical trial will be conducted at the dental clinic of the Universidade Nove de Julho*.* Patients will be divided by block randomization into three groups (*n* per group = 13), according to the treatment to be performed (Fig. 2). Opaque envelopes will be identified, and a sheet containing the information of the corresponding experimental group will be inserted. The envelopes will be sealed and will remain sealed in numerical order in a safe place until execution of the procedures.

### Participants

Students and employees from the Universidade Nove de Julho will be screened, and 39 subjects who fulfil the inclusion and exclusion criteria will be selected to participate in this research.

The inclusion criteria are as follows: patients of both genders, from 18 to 25 years old, who present a volatile sulphur compounds (SH_2_) ≥ 112 ppb measured using the Oral Chroma™.

The exclusion criteria are as follows: individuals with dentofacial anomalies (such as cleft lip or cleft palate), who are undergoing orthodontic and/or orthopaedic treatment, who have periodontal diseases (such as gingivitis or periodontitis) or dental cavities, who are undergoing oncological treatment, with systemic alterations (gastrointestinal, renal, hepatic), who have undergone antibiotic therapy up to 1 month before the survey, who are pregnant or who are smokers.

### Sample composition

To calculate the sample size, the data from the article by Costa da Mota et al. [21] were used. We established an error $err=\left|\overline{x_1}-\overline{x_2}\right|$, where $\overline{x_1}$ and $\overline{x_2}$ are the baseline values for periodontal treatment with aPDT. From this error, the effect size was calculated, given by:

$$ \frac{err}{\sqrt{\sigma_1^2+{\sigma}_2^2}} $$

where ${\sigma}_1^2$ and ${\sigma}_2^2$ are the variances of Groups 1 and 2, respectively.

Assuming that the studied groups have a normal or approximately normal distribution, that the sample size will be sufficiently large and that a two-tailed test will be used, for a significance level α = 0.05 and maintaining the power of the test 1 – β = 0.90 we have Group 1: 13 patients, Group 2: 13 patients, Group 3: 13 patients.

### Interventions

#### Tongue scraping

In Group 1, tongue scraping will be performed by the same operator in all patients. Posterior-anterior movements will be performed with a plastic scraper (Halicare®) over the lingual dorsum, followed by cleaning of the scraper with gauze. Also, the tip of the tongue will be cleaned with another piece of gauze in order to remove the tongue coating that could be accumulated in this area. This procedure will be performed ten times in each patient in order to standardize the mechanical removal of the coating. In the groups in which we use tongue scrapers (Groups 1 and 3), the scrapers will be given to the patients, and they will be instructed to use them every night. Data on whether they use the scraper regularly will be collected on the 7 days control, so the effect of tongue scraping can be checked as well as the compliance of patients to this technique.

#### Antimicrobial photodynamic therapy (aPDT)

Group 2 will be treated with aPDT. The LED light curing device Valo Cordless Ultradent®, an office appliance, with a coupled radiometer, and a spectrum of 440–480 nm will be used. Both the volunteer to be treated and the professional will be using specific eye protection glasses. The active end of the LED will be coated with clear disposable plastic (PVC), thus avoiding cross contamination. One session of aPDT with *Bixa orellana* extract in spray will be performed. *Bixa orellana* extract manipulated at a concentration of 20% (Fórmula e Ação®) will be applied in a sufficient amount to cover the middle third and back of the tongue. Following 2 min of pre-irradiation time, six points in the tongue dorsum with a distance of 1 cm between them will be irradiated. The apparatus will be pre-calibrated at wavelength 395–480 nm for 20 s and 9.6 J of energy per point, which is similar to the energy used in other protocols of aPDT in the tongue that have had satisfactory results [7, 15–17]. Table 1 shows all the parameters that shall be used. The energy of 9.6 J per point is similar to the energy used in other protocols of aPDT in the tongue that had satisfactory results [7, 15–17], which is why we are maintaining this parameter.

**Table 1:** LED parameters to be used in this study

Wavelength (nm)	395–480
Operating mode	Continuous wave
Average radiant power (mW)	480
Polarization	Random
Aperture diameter (cm)	0.9
Irradiance at aperture (mW/cm^2^)	762
Beam profile	Top hat
Beam spot size at target (cm^2^)	3.14
Irradiance at target (mW/cm^2^)	153
Exposure duration (s)	20
Radiant exposure (J/cm^2^)	6.37
Radiant energy (J)	9.6
Number of points irradiated	6
Area irradiated (cm^2^)	18.8
Application technique	Contact
Number and frequency of treatment sessions	1
Total radiant energy (J)	57.6

In Group 3, patients will receive tongue scraping performed by the same operator with instructions to perform the tongue scraping as described in Group 1, and also aPDT with *Bixa orellana* extract as described in Group 2.

### Outcome measures

#### Halimetry

Oral air collection will follow the Oral Chroma™ manufacturer’s guidelines. The participant will be instructed to rinse with a cysteine solution (10 mM) for 1 min and then remain with his/her mouth closed for 1 min. A syringe from the same manufacturer for collection of mouth air will be introduced into the patient’s mouth. For 30 s the patient will remain with his/her mouth closed, breathing through the nose, without touching the syringe with the tongue. The plunger will be pulled out, re-emptied into the patient’s mouth and pulled out again to fill the syringe with the breath sample. We will place the gas injection needle in the syringe and adjust the plunger to 0.5 ml. The collected gases will be injected into the entrance door of the unit in a single movement. This procedure will be carried out in all of the groups before treatment, immediately after treatment and after 7 days for comparison of the levels of VSCs.

To avoid changes in the halimetry, participants will be instructed to follow these instructions: 1. 48 h before evaluation, avoid ingesting food with garlic, onion or strong seasoning, alcohol consumption and the use of mouthwashes; 2. on the day of the evaluation, do not eat 2 h before the exam; abstain from coffee, candies, chewing gum and oral hygiene products; and perform teeth brushing only with water. In order to make the evaluations more similar, all patients will be scheduled in the afternoons, at approximately the same time, following the same instructions, so we can minimize variations. In addition, patients will be submitted to a brief anamnesis. Data will be collected about past and current health conditions, the use of dental floss, frequency of tooth brushing, tongue cleaning habits and the use of mouthwashes. These data will be collected in the baseline appointment and confirmed in the 7 days follow-up.

Two calibrated operators will also evaluate the presence of tongue coating. They will be trained and submitted to exercises in order to grade the presence of coating. These exercises will be conducted with 40 pictures, which will also be graded by a benchmark examiner who is specialized in halitosis and will provide the theoretical explanation before the training. The intra-examiner calibration will be performed with an interval of 2 days. A grade “0” will be awarded in cases where no coating can be seen; “1/3” in cases where only the posterior third of the tongue is covered with coating; “2/3” in cases where the posterior and middle thirds of the tongue are covered with coating; and “3/3” in cases where the whole dorsum of the tongue is covered with coating [28].

#### Microbiological analysis

The microbiological analysis of tongue coating will be performed by collecting biofilm samples from the region of the lingual dorsum with a 1-μl inoculation loop. Samples will be transferred into individual vials containing 1.5 ml of reduced transport fluid and placed in the vortex for approximately 30 s. After homogenization, the ten-fold dilution series will be prepared in 180 μl of sterile phosphate-buffered saline (PBS) solution and 10^− 2^, 10^− 3^, 10^− 4^ and 10^− 5^ aliquots and transferred to blood agar plates. Considering that the main bacteria responsible for the production of VSCs are Gram-negative and anaerobic, plaques will be incubated in an anaerobic jar for 72 h at 37 °C, for later counting of the colony-forming units (CFUs). This procedure will be performed before, immediately after and 7 days after treatment.

### Harms

The participants will be treated at the university, to make it easier for them to return, avoiding absences. No adverse effects are expected from any of the treatments.

### Statistical analysis

Data from Oral Chorma™ will be analysed for their normality by the Shapiro-Wilk test. If the normality hypothesis is accepted, the analysis of variance (ANOVA) followed by the Tukey test will be used when necessary to perform the between-groups analysis. To analyse the results of the treatment in both periods of the study, the *t* test for paired data will be used. If the normality hypothesis is rejected, the Kruskal-Wallis test followed by the Student-Newman-Keuls test, when necessary, will be used. To analyse the results of each treatment in both periods of the study, the Wilcoxon test will be used.

The kappa test will also be used to check interrater reliability in the evaluation of the presence of tongue coating.

## Discussion

Halitosis treatment is a topic that still needs attention, as it very often results in the social isolation of the patient and may eventually cause depression [29]. Studies have shown that self-cleaning of the tongue alone is not completely efficient for the reduction of halitosis, highlighting the importance of dental professionals in both treatment and prevention of this condition [9, 30]. Regarding the use of mouth rinses, there is limited evidence that they may be effective in the reduction of halitosis, and studies do not show a beneficial effect for the reduction of tongue coating [11]. Also, it has been suggested that the use of antimicrobial agents is associated with some collateral effects, such as unpleasant taste, staining effects and especially harm to normal oral microflora [31]. In contrast, aPDT has been proven as a good complementary treatment for this condition [7, 20–22]. As opposed to conventional antimicrobial methods, aPDT has the advantages of being a non-invasive procedure, it does not result in undesired side effects, and the development of resistance by bacteria is unlikely [15]. Additionally, one could speculate that patients also tend to appreciate new and technological methods of treatment, which could increase the satisfaction regarding this therapy.

To date, studies using aPDT have been carried out using methylene blue in combination with a red laser. *Bixa orellana* is a natural, innovative and reddish PS, which allows its use with blue LEDs and could turn this protocol of treatment into a more accessible one, since the majority of dentists already have this source of light in their offices. Therefore, the present study could be of great importance to clarify if the application of aPDT with a blue LED and *Bixa orellana* results in the same trend of reduction of halitosis as that reported in the literature when using the combination of treatments [13, 14].

The period of 7 days for control was established in order to verify the recolonization of bacteria, which usually happens very quickly in the tongue. The results immediately after the different treatments are the most important ones, as they will really show their efficacy. Additionally, the investigations using aPDT for the treatment of halitosis show its effects immediately after treatment [7, 21, 22]. Furthermore, this trial was designed to be applied in the dental practice in the future, so the 7-day interval also represents a period in which patients could come back to the dental office, retake the halimetry and repeat the aPDT, if necessary. This way, dentists will be able to control the presence of halitosis and treat patients once a week in persistent cases. This protocol will also enable the comparison of aPDT alone with tongue scraping alone and with the combination of both techniques in order to verify the most efficient treatment.

It is also important to point out that, while halitosis is an important issue for young people, most studies on this subject are conducted in older patients. Moreover, tongue coating seems to be the principle cause of halitosis in this population [5]. In conclusion, it is crucial to study the age range in question in this protocol and possible treatments to reduce the presence of tongue coating in young adults.

### Trial status

This study is not yet recruiting participants.

## Additional file

Additional file 1: SPIRIT 2013 checklist: recommended items to address in a clinical trial protocol and related documents. (DOC 121 kb)

## Abbreviations

aPDT: Antimicrobial photodynamic therapy
CFU: Colony-forming unit
LED: Light-emitting diode
SH_2_: Sulphide
SPIRIT: Standard Protocol Items: Recommendations for Interventional Trials
VSCs: Volatile sulphur compound
